# Supersensitive Odorant Receptor Underscores Pleiotropic Roles of Indoles in Mosquito Ecology

**DOI:** 10.3389/fncel.2018.00533

**Published:** 2019-01-24

**Authors:** David M. Ruel, Esther Yakir, Jonathan D. Bohbot

**Affiliations:** Department of Entomology, The Hebrew University of Jerusalem, Rehovot, Israel

**Keywords:** odorant receptor, indole, skatole, mosquito, *Aedes aegypti*

## Abstract

Mosquitoes exhibit highly diverse and fast evolving odorant receptors (ORs). The indole-sensitive OR gene clade, comprised of *Or2* and *Or10* is a notable exception on account of its conservation in both mosquito subfamilies. This group of paralogous genes exhibits a complex developmental expression pattern in *Aedes aegypti*: *AaegOr2* is expressed in both adults and larvae, *AaegOr10* is adult-specific and a third member named *AaegOr9* is larva-specific. OR2 and OR10 have been deorphanized and are selectively activated by indole and skatole, respectively. Using the two-electrode voltage clamp of *Xenopus* oocytes expressing *Ae*. *aegypti* ORs, we show that AaegOR9 is supersensitive and narrowly tuned to skatole. Our findings suggest that *Ae*. *aegypti* has evolved two distinct molecular strategies to detect skatole in aquatic and terrestrial environments, highlighting the central ecological roles of indolic compounds in the evolutionary and life histories of these insects.

## Introduction

Aromatic and heterocyclic compounds play an important role in the ecology of adult mosquitoes as indicated by the odor space of the *Anopheles gambiae* odorant receptor (OR) repertoire (Carey et al., 2010; Wang et al., 2010). Specifically, indole (IUPAC name, 1H-indole) and skatole (IUPAC name, 3-methylindole) are respectively detected by the narrowly tuned *Or2* and *Or10* paralogous genes found in *Culex quinquefasciatus* (Hughes et al., 2010; Pelletier et al., 2010), *Aedes aegypti* and *An. gambiae* (Carey et al., 2010; Wang et al., 2010) reflecting their ancestral origin (Bohbot et al., 2011). Due to the sensitive and selective nature of the OR2-indole and OR10-skatole interactions, they have been referred to as the “indolergic” receptors (Bohbot and Pitts, 2015).

Indole and skatole are released by a wide variety of organisms but are mainly synthesized by bacteria (Elgaali et al., 2002; Schulz and Dickschat, 2007; Lindh et al., 2008; Hubbard et al., 2015), fungi (Chen et al., 2014; Tomberlin et al., 2017) and plants (Turlings et al., 1991; Frey et al., 2000; Ober, 2005). In adult mosquitoes, both compounds have been proposed to mediate oviposition site (Blackwell and Johnson, 2000) and host-locating behaviors (Cork, 1996). However, their exact ecological role(s) remain complex since indoles are major constituents of floral (Knudsen et al., 2006) and animal scents (Meijerink et al., 2001; Lee et al., 2015). Interestingly, indolic compounds play additional ecological roles in mosquito larvae (Xia et al., 2008; Scialò et al., 2012).

*Or2* is expressed in the adult and larval stages of *Ae. aegypti* (Bohbot et al., 2007) and *An. gambiae* (Hill et al., 2002; Xia et al., 2008). *Or10* expression is more complex: in *An. gambiae*, *Or10* is expressed both in larvae and adults. In *Ae. Aegypti*, *Or10* is only expressed in adults, while a third paralog named *Or9*, is expressed in the larval antenna (Bohbot et al., 2007). Based on pharmacological studies, we have suggested that receptor sensitivity towards odorants in the nanomolar concentration range is a predictor of OR-semiochemical relationships (Bohbot and Pitts, 2015). The activation of AaegOR9 by indole in the low micromolar concentration range (Bohbot et al., 2011) indicated that a more potent indolic cognate ligand selectively activates this receptor.

Using a reverse chemical ecology approach, we set out to identify a potential cognate ligand for this larval-expressed *Or9* gene (Supplementary Table 1). First, we used a panel of 31 indole derivatives from plants and microbes to identify a potent activator of AaegOR9, then we showed that AaegOR9 is narrowly tuned to skatole in the low nanomolar concentration range. Our findings suggest that Culicinae have developed a supersensitive skatole receptor that operates in water where this compound exhibits low solubility. The occurrence of two skatole receptors, each assigned to a different developmental stage indicates the central role of this odorant in the *Ae. aegypti* life cycle. The deorphanization of AaegOR9: (i) provides a molecular target for future larval behavioral disruption studies; (ii) improves our understanding of insect OR coding; and (iii) raises questions on the possible ecological roles of mosquito indolergic receptors.

## Materials and Methods

### Chemical Reagents

The chemicals (Supplementary Table 1) used for the deorphanization of AaegOR9 were obtained from Sigma-Aldrich (Milwaukee, WI, USA), ChemCruz (Dallas, TX, USA), Glentham Life Sciences (Corsham, UK), FluoroChem (Hadfield, UK), SL Moran (Jerusalem, Israel), Holland Moran (Yehun, Israel), Alfa Aesar (Ward Hill, MA, USA) and from the generous contribution of the Dr. Kolodkin-Gal Lab (Weizmann Institute of Science, Israel).

### Two-Electrode Voltage Clamp of *Xenopus* Oocytes Expressing ORs

The methodologies and protocols have been described in details elsewhere (Bohbot and Dickens, 2009). *AaegOr9* and *Aaeg-ORco* cRNAs (Bohbot et al., 2011) were synthesized from linearized pSP64DV expression vectors using the mMESSAGE mMACHINE^®^ SP6 kit (Life Technologies). Stage V-VII oocytes were harvested from *Xenopus* *laevis* females, mechanically separated, treated with collagenase (8 mg/mL, 30 min, 18°C) and rinsed in washing solution (96 mM NaCl, 2 mM KCl, 5 mM MgCl_2_ and 5 mM HEPES, pH 7.6). Oocytes were microinjected with 27.6 ng *AaegOr9* and *AaegORco* cRNAs, incubated at 18°C for 3–4 days in ND96 solution (96 mM NaCl, 2 mM KCl, 5 mM MgCl_2_, 0.8 mM CaCl_2_ and 5 mM HEPES, pH 7.6), supplemented with 5% dialyzed horse serum, 50 μg/mL tetracycline, 100 μg/mL streptomycin and 550 μg/mL sodium pyruvate. Whole-cell currents were recorded using the two-microelectrode voltage-clamp technique. During recording sessions, the holding potential was maintained at −80 mV using an OC-725C oocyte clamp (Warner Instruments, LLC, Hamden, CT, USA). Oocytes placed in a RC-3Z oocyte recording chamber (Warner Instruments, LLC, Hamden, CT, USA) were exposed to odorants for 8 s. Current was allowed to return to baseline between odorant applications. Data acquisition and concentration-response analyses were carried out with a Digidata 1550A and the pCLAMP10 software (Molecular Devices, Sunnyvale, CA, USA), and analyzed using GraphPad Prism 7 (GraphPad Software Inc., La Jolla, CA, USA). Stock concentration of odorants (10^−2^ M) were dissolved in ringer solution containing 2% dimethyl sulfoxide (DMSO) in order to solubilize the hydrophobic indolic compounds.

### Pharmacological Characterization

The response profile was established using multiple sessions, each including six compounds at a time and indole as an internal reference. The order in which these compounds were administered was reversed within a session to mitigate against any potential sequence effects between compounds (none were observed). All the response values were normalized to the indole reference in each recording session (Supplementary Figure 1).

For the establishment of the concentration-response curves, oocytes were exposed to increasing concentrations of indole, skatole and indole-3-carboxaldehyde (I3C; Supplementary Figure 2). Quantitative characterization of OR sensitivity was estimated using the averaged effective concentration at 50% of the maximal response (EC_50_) over the sample population. The data to establish the concentration response curve and EC_50_ of AaegOR10-skatole was extracted from a previous study (Bohbot and Dickens, 2012).

### Phylogeny OR Intron-Exon Structure and Phylogeny

All the sequences used in our phylogenic analysis were obtained from the VectorBase and NCBI databases using *AaegOr2*/*9*/*10* as query (for accession numbers, see Supplementary Table 3). DNA sequences for *Toxorhynchites* *Or2* and *Or10* can be accessed here: http://dx.doi.org/10.6084/m9.figshare.1092617. MAFFT version 7 (Nakamura et al., 2018) was used for multiple amino-acid sequence alignment. The phylogenic software IQ-TREE (Nguyen et al., 2015; Kalyaanamoorthy et al., 2017; Hoang et al., 2018) and the FigTree editor^1^ were used for building the mosquito indolergic receptor phylogenic tree based on the maximum likelihood method (Model: JC, UFbootstrap: 5,000). Using MAFFT (default parameters) and the Vectorbase database, we located the intron positions on the indolergic receptor genes.

## Results

### AaegOR9 Is Narrowly Tuned to Skatole

Based on its larval expression (Bohbot et al., 2007) and functional characterization (Bohbot et al., 2011), we initially surmised that AaegOR9 would be narrowly tuned to a water-soluble indolic ligand. To test this hypothesis, we screened AaegOR9 with a panel of 31 indolic derivatives (Supplementary Table 1) exhibiting some degree of water solubility using the two electrodes voltage clamp of *Xenopus* *laevis* oocytes. We also included indole and skatole as references (Bohbot et al., 2011). The screen was carried out using a low odorant concentration (500 nM) in order to mitigate the caveats associated with high ligand concentrations, including receptor adaptation, antagonist effects and technical artifacts such as broad molecular receptive ranges (Bohbot and Pitts, 2015).

**Figure 1:**
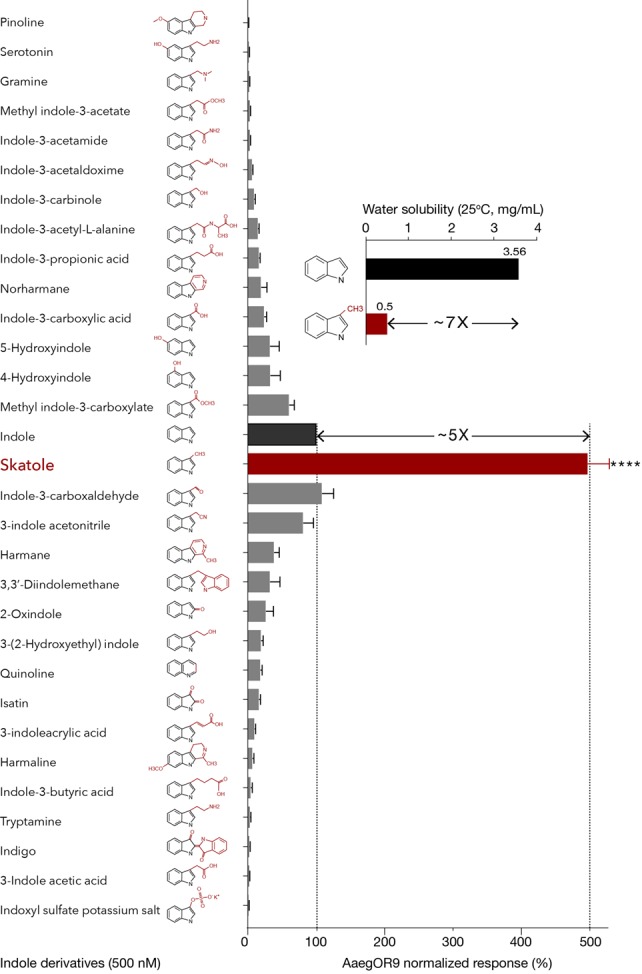
*Aedes aegypti* OR9 (AaegOR9) is narrowly tuned to skatole. Indole-tuning curve of AaegOR9 to an odor panel comprised of 31 indole derivatives (kurtosis value: 17.75). Skatole is five times more potent than indole (*t*-test: ****p < 0.0001; *n* = 10; 100% and 500% response thresholds shown as dotted lines), which is inversely correlated with their respective water solubility (inset). Error bars of average responses indicate standard errors.

At this concentration, skatole was decisively the most potent ligand, eliciting responses five times higher than indole (Figure 1) confirming the hypothesis that the potential cognate ligand of AaegOR9 is an indole derivative. However, this result contradicts our water-soluble ligand hypothesis. Indeed, skatole is about seven times less water soluble (0.5 mg/mL, ChemID*plus*) than indole (3.56 mg/mL, ChemID*plus*; Figure 1) and is considered rather insoluble in water as a result.

I3C, indole, 3-indole acetonitrile and methyl indole-3-carboxylate were the next most potent ligands suggesting that indole derivatives with a short side chain on position C3 can fit into the binding pocket of AaegOR9. However, comparable small indolic compounds such as indole-3-carbinole or gramine were among the least potent ligands (Figure 1). Overall, the AaegOR9 response profile was narrow (kurtosis value of 17.75), especially considering that our odorant panel was restricted to indole derivatives.

### AaegOR9 Is Supersensitive to Skatole

**Figure 2:**
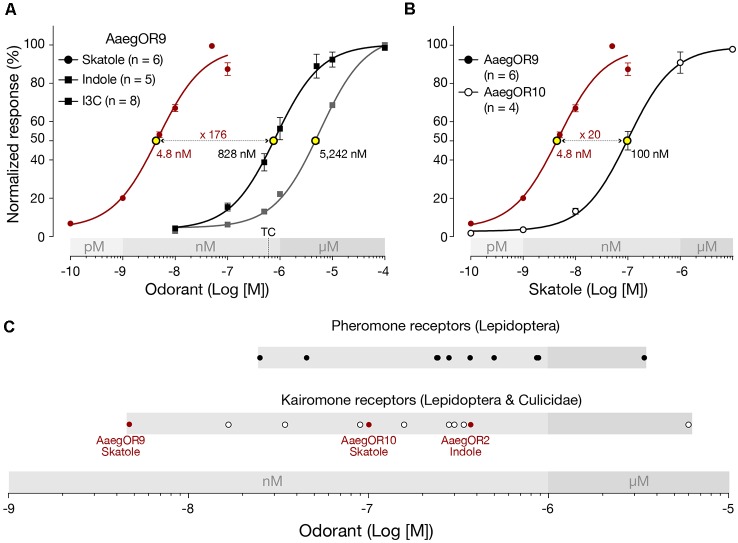
*Aedes aegypti* OR9 (AaegOR9) is a supersensitive skatole receptor. **(A)** Based on their respective EC_50_ values (yellow dots), AaegOR9 is significantly (one-way ANOVA followed by Tukey’s post test; *p* < 0.0001) more sensitive to skatole than to indole or to indole-3-carboxaldehyde (I3C). The concentration (500 nM) to which the tuning curve is based on is indicated by “TC.” **(B)** AaegOR9 is a more sensitive skatole receptor than AaegOR10 (*t*-test; *p* < 0.01). **(C)** Sensitivity ranking (according to EC_50_ values of cognate receptor-semiochemical interactions) of pheromone and kairomone receptors (Supplementary Table 2).

To characterize the pharmacological sensitivity of AaegOR9, we measured the amplitudes of the current responses of this receptor when exposed to increasing concentrations (100 pM to 10 μM) of skatole, indole and I3C (Figure 2A). Compound sensitivities were determined using the extrapolated EC_50_ values. This analysis confirmed that AaegOR9 is 176 times more sensitive to skatole than to indole. AaegOR9 is a significantly more sensitive skatole receptor (EC_50_ ≃ 5 nM) than AaegOR10 (EC_50_ = 100 nM; Figure 2B). Although I3C and indole elicited comparable responses in our tuning curve experiment (Figure 1), their EC_50_ values were significantly different (Figure 2A), underscoring the caveat of inferring receptor sensitivity based on tuning curves.

Plotting the EC_50_ values of AaegOR9 against previously characterized OR-cognate odorant pairs (Supplementary Table 2) using our pharmacological platform (Bohbot and Pitts, 2015) reveals that the AaegOR9-skatole pair is the most sensitive indolergic OR (Figure 2C) and the most sensitive OR-ligand pair identified so far, outperforming the most sensitive pheromone receptor.

### OR9 Is a Culicinae-Specific Gene Expansion

**Figure 3:**
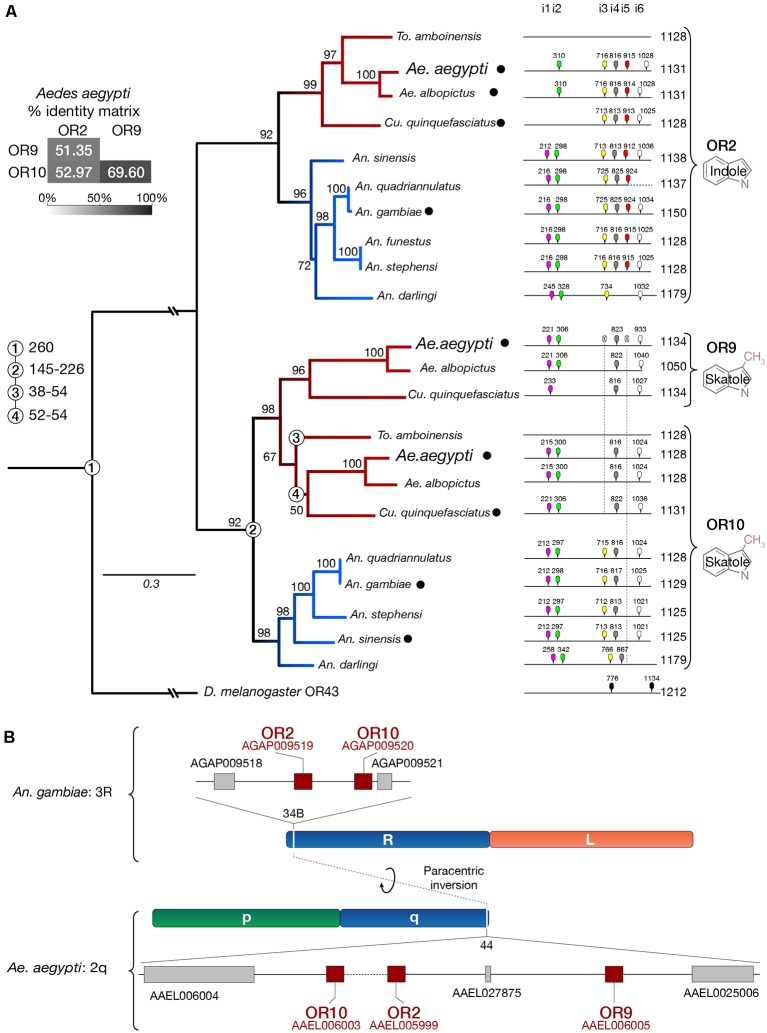
*Or9* is a Culicinae-specific gene expansion. **(A)** DNA sequence identity, substitution rates, intron locations and odorant ligands (deorphanized receptors are labeled with a black dot, see Supplementary Table 2) suggest that *Or9* is a Culicinae-specific gene expansion while *Or2* and *Or10* are present in both Culicinae (red branches) and Anophelinae (blue branches). Intron locations are color-coded and numbered from 1 to 6 (i1–i6). Missing introns are indicated by a crossed intron with a dotted lines underneath. Bootstrap values (%) are based on 5,000 replicates. Numbered circles on branch points indicate lineage splits in million years (MY). **(B)** Indolergic receptors are located on the q arm of chromosome 2 and on the R arm of chromosome 3 in *Ae*. *aegypti* and *An*. *gambiae*, respectively. Transcript numbers are shown for *An. gambiae* (AGAP#) and *Ae. aegypti* (AAEL#).

The conserved *Or2*, O*r9*, *Or10* genes were initially identified from the *An. gambiae* and *Ae. aegypti* genomes (Hill et al., 2002; Bohbot et al., 2007, 2011). These genes encode proteins with high amino-acid sequence identity considering the sequence divergence characteristic of this family (Figure 3A). We have extended our previous analysis (Bohbot et al., 2011) by including additional *Anopheles* species, *Ae. albopictus* and *Toxorhynchites amboinensis*. We confirm that the indolergic receptor clade is divided into the OR2 and OR10 subgroups indicating their ancestral origin (Figure 3A). *Or9* emerged in the Culicinae lineage 52–54 mya (Arensburger et al., 2010), which includes *Ae. albopictus* and *Cu. quinquefasciatus*. There is no information regarding Or expression in *To. amboinensis*. We failed to identify any signature of other paralogs in *An. gambiae* and *Ae. aegypti* consistent with the hypothesis that the *Or9*-*Or10* split occurred in Culicinae.

The greater similarity between AaegOR9 and AaegOR10 not only includes sequence conservation and amino-acid substitution rates but is also reflected by the patterns of conserved introns (Figure 3A). Both Culicinae *Or10* and *Or9* genes are missing introns 3 and 5. The absence of intron 5 is also a conserved feature within the Anophelinae *Or10* clade. *Or2*, O*r9*, *Or10* are clustered together in region 44 of the q arm of chromosome 2 in *Ae. aegypti* (Figure 3B). This region corresponds to region 34B of the orthologous chromosome 3R in *An. gambiae*, reflecting the extensive paracentric inversion events that occurred within chromosome segments between these two species (Severson et al., 2004; Arensburger et al., 2010).

## Discussion

We had originally reported AaegOR9 as a broadly tuned receptor sensitive to indole in the low micromolar range suggesting that its cognate ligand, likely an indole derivative, remained to be identified (Bohbot et al., 2011). Here, we have shown that skatole selectively and reversibly activates AaegOR9 in the high picomolar (below 1 nM) to low nanomolar (between 1 and 10 nM) concentration range consistent with the idea that it is the cognate odorant ligand for this receptor (Bohbot and Pitts, 2015).

**Figure 4:**
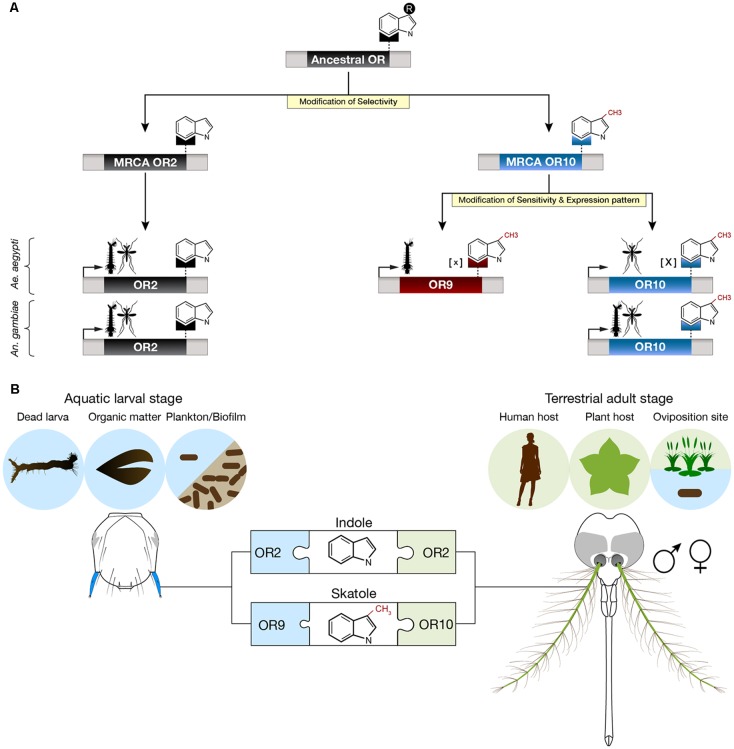
Proposed evolution and ecological roles of *Aedes aegypti* indolergic receptors. **(A)** The *Or2*, *9* and *10* gene clade derives from two successive gene duplication events followed by neofunctionalization (yellow highlights) consisting of modifications of ligand selectivity, sensitivity and developmental expression patterns. The most parsimonious hypothesis is that the most recent common ancestors (MRCAs) of *Or2* and *Or10* derived from a gene duplication event prior to the Anophelinae-Culicinae split. This event lead to neofunctionalization by means of selective detection of indole derivatives. The second duplication event arose in the Culicinae subfamily (e.g., *Ae. aegypti*) leading to the emergence of a low [X] and high [x] sensitivity skatole receptor, each expressed in a distinct developmental stage. **(B)** Putative ecological roles of indole and skatole in adults and larvae. Adults may detect both indole compounds to identify suitable hosts and oviposition sites while larvae may use these compounds to locate food sources, including dead larvae, decomposing organic matter and microbes.

Based on our analyzes and on the principle of parsimony, we propose that *Or2*, *Or9* and *Or10* derive from two gene duplication events, one preceding the Anophelinae-Culicinae split that occurred 145–226 mya (Krzywinski et al., 2006; Reidenbach et al., 2009) and a second one that occurred in the Culicinae lineage (Figure 4A). The most common recent ancestors of *Or2* and *Or10* were likely indole and skatole receptors since this function is still conserved in the two mosquito subfamilies (Bohbot et al., 2007). We hypothesize, that the original ancestral receptor was sensitive to an indolic compound, perhaps indole or skatole. Although it is unlikely, due to the nanomolar sensitivity of OR10 for skatole, it is conceivable that the cognate ligand for OR10 remains to be identified. It is remarkable that during their evolution, mosquitoes have retained the function of discriminating between close structural chemical analogs, differing by a methyl group on position C3.

While both AaegOR10 and AaegOR9 act as skatole receptors, they have diverged in function: larval AaegOR9 has a significantly higher sensitivity for this compound than adult AaegOR10. In addition, the expression pattern of *AaegOr10* and *AaegOr9* also diverged, allocating the role of skatole detection to the former in adults and to the latter in larvae (Figure 4A). It is interesting that other insects, including *Drosophila melanogaster* larvae and adults detect the same cues using different ORs (Dweck et al., 2015). In our case, the role for this increased sensitivity and distinct expression patterns remain unclear.

Skatole seems to be playing a unique role in *Ae. aegypti*. The increased sensitivity of OR9 to skatole may correspond to an evolutionary adaptation to the reduced water solubility of this compound, conferring a fitness advantage at the larval stage. Being outperformed, larval *Or10* expression may have been relegated to the adult stage where skatole can occur at much higher concentrations due to its high volatility. It is interesting that both larva and adult *An. gambiae* detect skatole using the same receptor. Considering the differential sensitivity and developmental expression pattern of *AaegOr9* and *AaegOr10*, we do not think this is a case of gene redundancy.

During the terrestrial stage, adult *Ae. aegypti* use olfaction to locate nectar sources, suitable oviposition sites and animal hosts using about 80 *Or* genes. The aquatic larva has a more limited range of behaviors (mostly feeding and escape behaviors), occupy one type of habitat and express 23 *Or* genes of which 15 are larval specific. Based on our study, it will be interesting to explore whether other larval-specific receptors have evolved enhanced sensitivity to poorly soluble volatile organic compounds.

Larvae mainly graze on biofilm (fungi, bacteria, algae), dead larvae (Kinney et al., 2014) and other decomposing organic matter including plant materials, which are sources of indolic compounds (Figure 4B). The exact roles of indole in adult mosquitoes, while traditionally ascribed to oviposition (Blackwell and Johnson, 2000), is much more complex than previously thought as male adult mosquitoes, as well as larvae, have relied on these compounds in the last 200 millions of years of evolution. These findings underscore the conserved and different roles indoles assume in the life histories of mosquitoes. It now remains to untangle the physiological, behavioral and ecological roles of these interesting compounds in the life of these insects.

## Data Availability

The datasets generated for this study can be found in Figshare, http://dx.doi.org/10.6084/m9.figshare.1092617.

## Ethics Statement

All applicable international, national, and/or institutional guidelines for the care and use of animals were followed (NIH approval number: OPRR-A01-5011).

## Author Contributions

JB designed and wrote the manuscript. EY provided technical support. DR carried out the experiment, analyzed the data and participated in the writing of the manuscript.

## Conflict of Interest Statement

The authors declare that the research was conducted in the absence of any commercial or financial relationships that could be construed as a potential conflict of interest.

## Footnotes

^1^http://tree.bio.ed.ac.uk/software/figtree/

## Supplementary Material

FIGURE S1 Examples of current traces of AaegOR9-ORco in response to 500 nM of 31 indole derivatives.

FIGURE S2 Examples of current traces of AaegOR9-ORco in response to increasing concentrations (Log units) of indole, skatole and indole-3-carboxaldehyde.

TABLE S1 Indole derivatives ranked according to their potency (see Figure 1).

TABLE S2 List of cognate pheromone and kairomone receptors deorphanized in the *Xenopus laevis* and other functional bioassays.

TABLE S3 Accession numbers of indolergic receptors used in Figure 3 (*Ae*., *Aedes*; *Cu*., *Culex*; *An*., *Anopheles*; *To*., *Toxorhynchites*; *D*., *Drosophila*).
