# Corrigendum: Supersensitive Odorant Receptor Underscores Pleiotropic Roles of Indoles in Mosquito Ecology

**DOI:** 10.3389/fncel.2019.00488

**Published:** 2019-11-06

**Authors:** David M. Ruel, Esther Yakir, Jonathan D. Bohbot

**Affiliations:** Department of Entomology, The Hebrew University of Jerusalem, Rehovot, Israel

**Keywords:** odorant receptor, indole, skatole, mosquito, *Aedes aegypti*

An author name was incorrectly provided as “David Ruel.” The correct name is “David M. Ruel.” A correction has been made to the author list.

Furthermore, in the original article, there was a mistake in Figure 2 as published. There is a typo in the legend of the y axis in Graph A. “Normalized esponse (%)” should be “Normalized response (%).” The corrected Figure 2 appears below.

**Figure 2:**
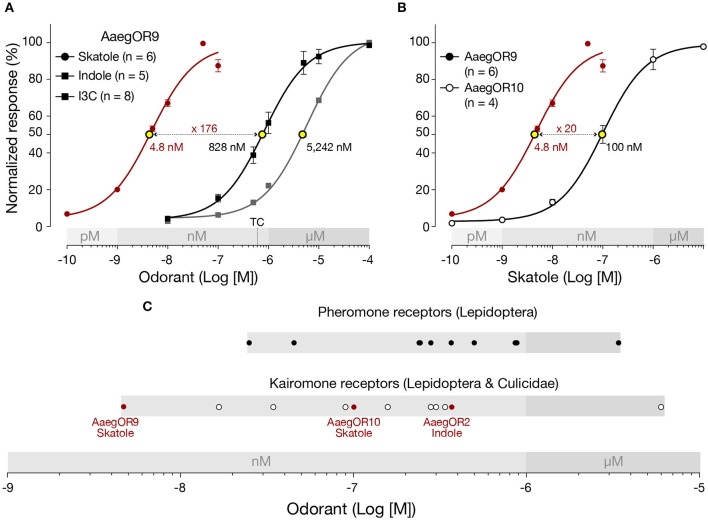
*Aedes aegypti* OR9 (AaegOR9) is a supersensitive skatole receptor. **(A)** Based on their respective EC_50_ values (yellow dots), AaegOR9 is significantly (one-way ANOVA followed by Tukey's post test; *p* < 0.0001) more sensitive to skatole than to indole or to indole-3-carboxaldehyde (I3C). The concentration (500 nM) to which the tuning curve is based on is indicated by “TC.” **(B)** AaegOR9 is a more sensitive skatole receptor than AaegOR10 (*t*-test; *p* < 0.01). **(C)** Sensitivity ranking (according to EC_50_ values of cognate receptor-semiochemical interactions) of pheromone and kairomone receptors (Supplementary Table 2).

Lastly, in the original article, there was a mistake in Figure 3 as published. The proposed exon structure for OR2 does not fit the phylogenetic tree labels. Aedine and Anopheline have erroneously been swapped. The corrected Figure 3 appears below.

**Figure 3:**
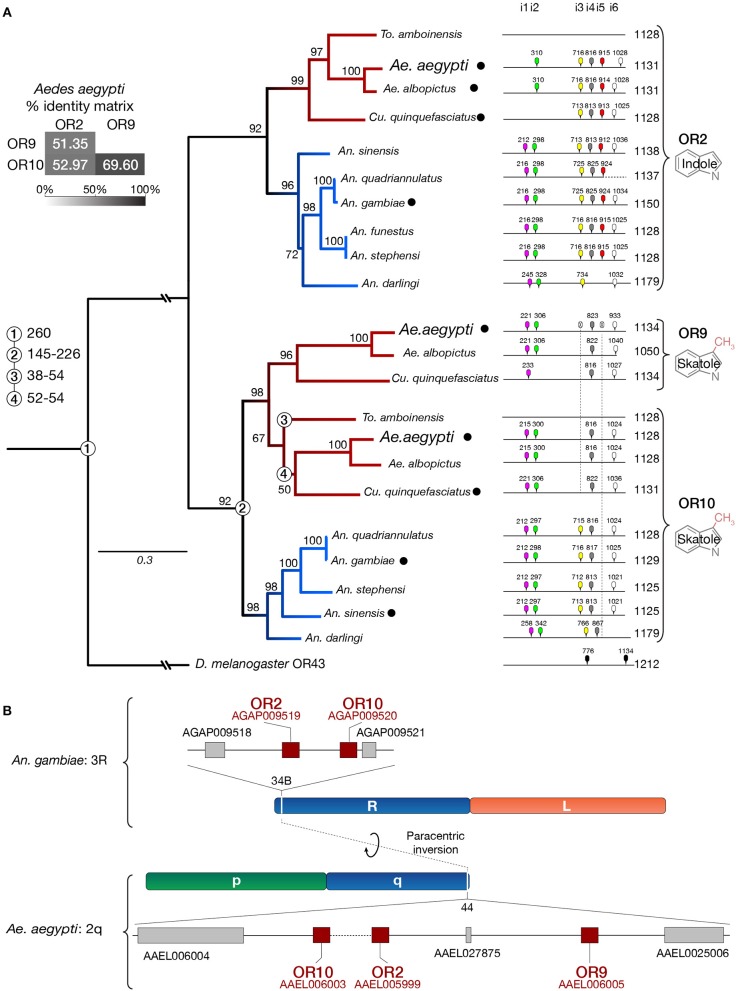
*Or9* is a Culicinae-specific gene expansion. **(A)** DNA sequence identity, substitution rates, intron locations and odorant ligands (deorphanized receptors are labeled with a black dot, see Supplementary Table 2) suggest that *Or9* is a Culicinae-specific gene expansion while *Or2* and *Or10* are present in both Culicinae (red branches) and Anophelinae (blue branches). Intron locations are color-coded and numbered from 1 to 6 (i1–i6). Missing introns are indicated by a crossed intron with a dotted lines underneath. Bootstrap values (%) are based on 5,000 replicates. Numbered circles on branch points indicate lineage splits in million years (MY). **(B)** Indolergic receptors are located on the q arm of chromosome 2 and on the R arm of chromosome 3 in *Ae*. *aegypti* and *An*. *gambiae*, respectively. Transcript numbers are shown for *An. gambiae* (AGAP#) and *Ae. aegypti* (AAEL#).

The authors apologize for these errors and state that this does not change the scientific conclusions of the article in any way. The original article has been updated.

