# Supplementary figures and images for: Supersensitive Odorant Receptor Underscores Pleiotropic Roles of Indoles in Mosquito Ecology
Source: Front Cell Neurosci. 2019 Jan 24;12:533. doi: 10.3389/fncel.2018.00533 (PMC6353850; doi:10.3389/fncel.2018.00533)

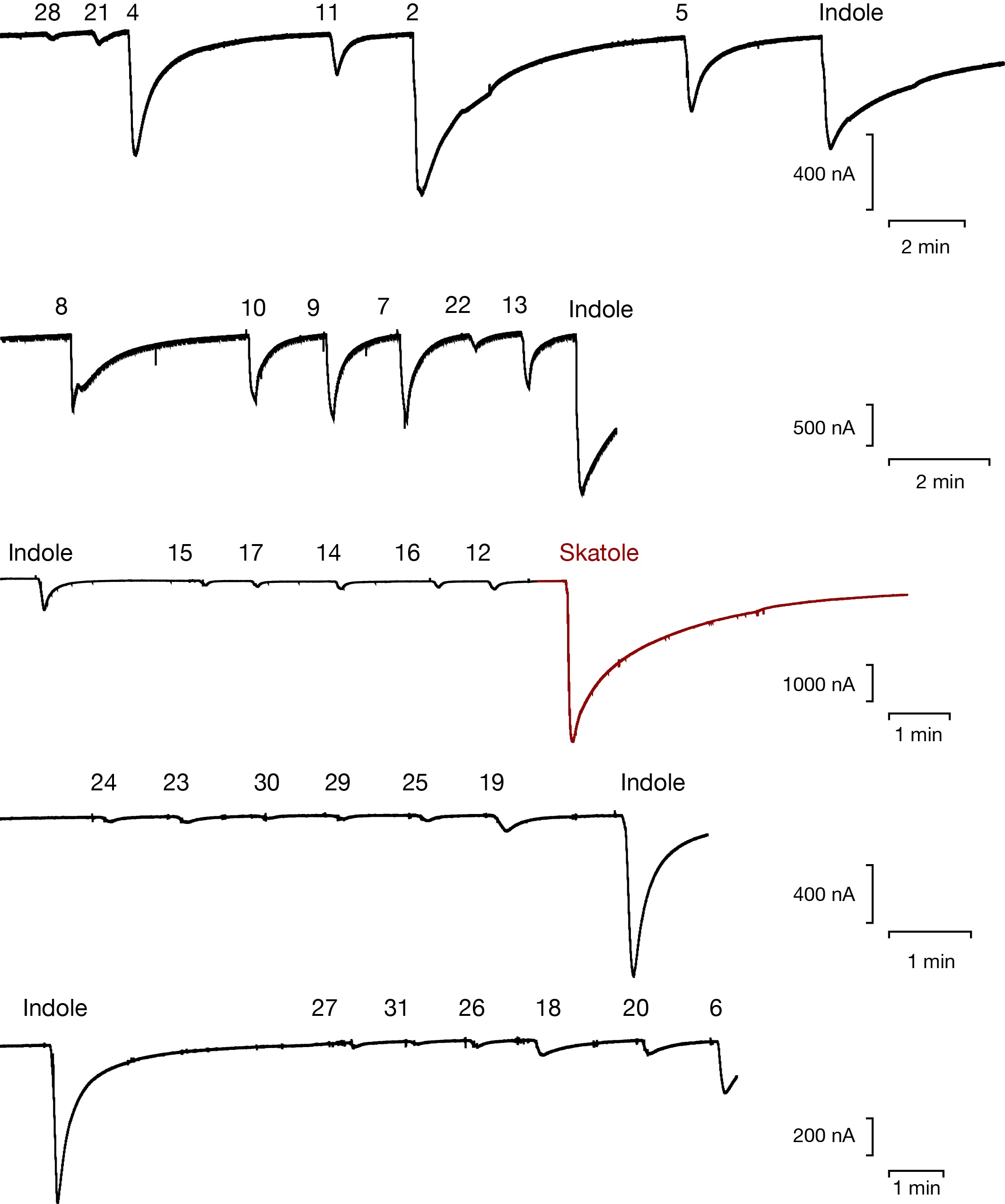

Supplement: FIGURE S1 — Examples of current traces of AaegOR9-ORco in response to 500 nM of 31 indole derivatives. [file Image_1.JPEG]

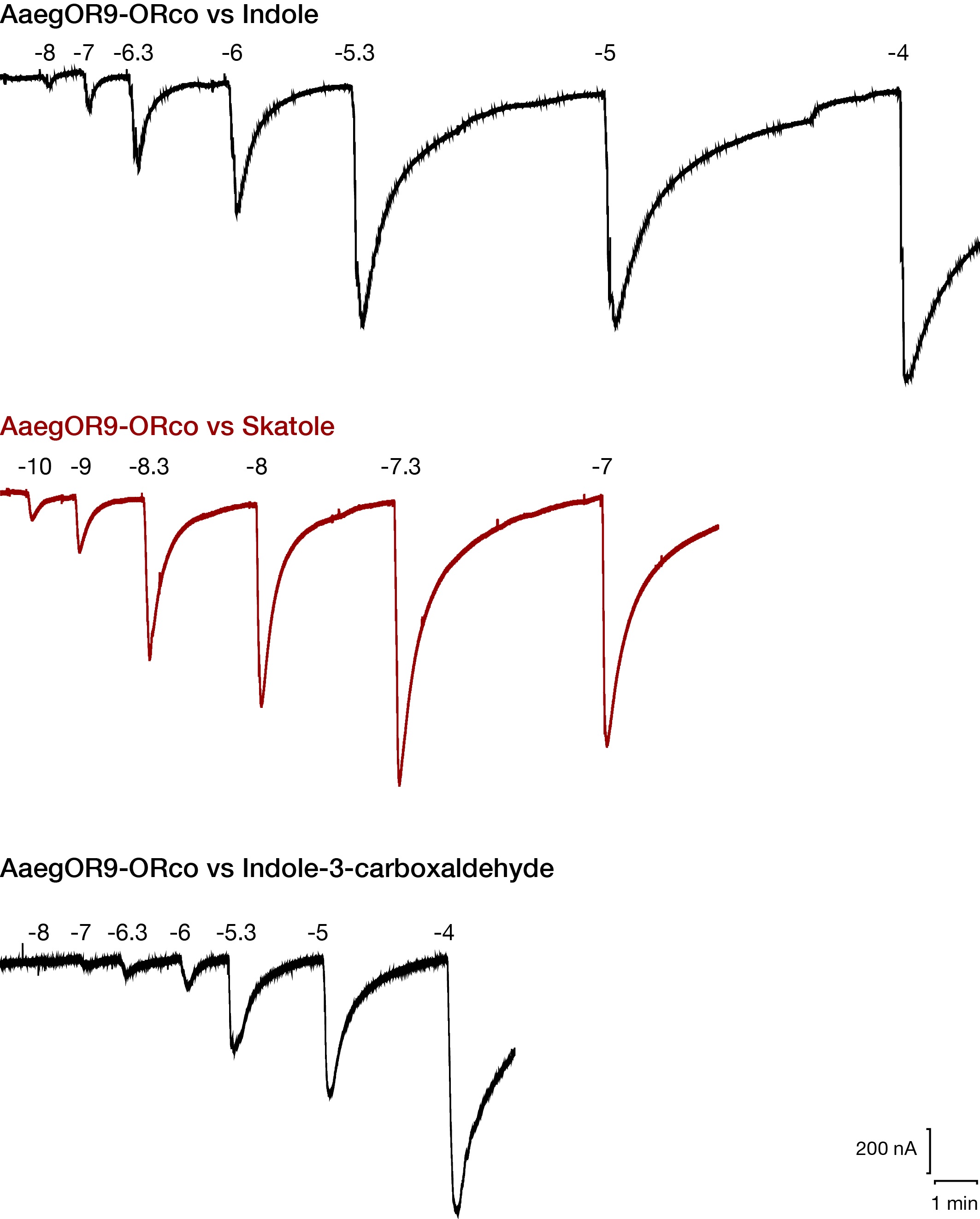

Supplement: FIGURE S2 — Examples of current traces of AaegOR9-ORco in response to increasing concentrations (Log units) of indole, skatole and indole-3-carboxaldehyde. [file Image_2.JPEG]
