# Supplementary material for: Supersensitive Odorant Receptor Underscores Pleiotropic Roles of Indoles in Mosquito Ecology
Source: Front Cell Neurosci. 2019 Jan 24;12:533. doi: 10.3389/fncel.2018.00533 (PMC6353850; doi:10.3389/fncel.2018.00533)
Supplement: TABLE S1 — Indole derivatives ranked according to their potency (see Figure 1). [file Table_1.pdf]

Supplementary table 1. Indole derivatives ranked according to their potency (see figure 1).

No.	Indole derivative	CAS No.	Structural formula	Supplier
1	3-Methylindole (skatole)	83-34-1	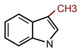	Sigma-Aldrich
2	Indole-3-carboxaldehyde	487-89-8	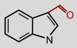	Dr. Kolodkin-Gal Lab
3	Indole	120-72-9	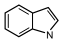	Sigma-Aldrich
4	3-indole acetonitrile	771-51-7	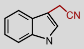	Dr. Kolodkin-Gal Lab
5	Methyl indole-3-carboxylate	942-24-5	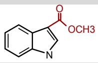	Dr. Kolodkin-Gal Lab
6	Harmane	486-84-0	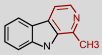	ChemCruz
7	4-Hydroxyindole	2380-94-1	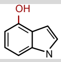	Sigma-Aldrich
8	3,3'-Diindolemethane	04-05-68	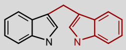	SL Moran
9	5-Hydroxyindole	1953-54-4	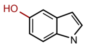	FluoroChem
10	2-Oxindole	59-48-3	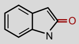	SL Moran
11	Indole-3-carboxylic acid	771-50-6	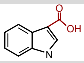	Dr. Kolodkin-Gal Lab
12	3-(2-Hydroxyethyl) indole	526-55-6	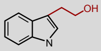	Dr. Kolodkin-Gal Lab
13	Norharmane	244-63-3	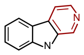	Sigma-Aldrich
14	Quinoline	91-22-5	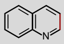	FluoroChem
15	Indole-3-propionic acid	830-96-6	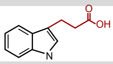	SL Moran
16	Isatin	91-56-5	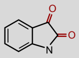	FluoroChem
17	Indole-3-acetyl-L-alanine	57105-39-2	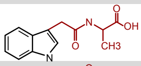	Sigma-Aldrich
18	3-Indoleacrylic acid	1204-06-4	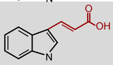	FluoroChem
19	Indole-3-carbinole	700-06-1	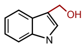	Sigma-Aldrich
20	Harmaline	304-21-2	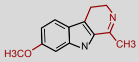	Glentham Life Sciences
21	Indole-3-acetaldoxime	09-06-76	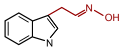	Dr. Kolodkin-Gal Lab
22	Indole-3-butyric acid	133-32-4	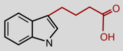	Sigma-Aldrich
23	Indole-3-acetamide	879-37-8	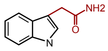	SL Moran
24	Tryptamine	61-54-1	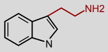	FluoroChem
25	Methyl indole-3-acetate	1912-33-0	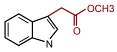	SL Moran
26	Indigo	482-89-3	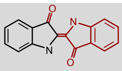	Glentham Life Sciences
27	Gramine	87-52-5	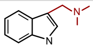	Holland Moran
28	3-Indole acetic acid	87-51-4	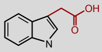	Dr. Kolodkin-Gal Lab
29	Serotonin	50-67-9	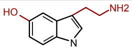	Sigma-Aldrich
30	Indoxyl sulfate potassium salt	2642-37-7	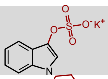	Alfa Aesar
31	Pinoline	20315-68-8	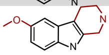	ChemCruz
