# Supplementary material for: Supersensitive Odorant Receptor Underscores Pleiotropic Roles of Indoles in Mosquito Ecology
Source: Front Cell Neurosci. 2019 Jan 24;12:533. doi: 10.3389/fncel.2018.00533 (PMC6353850; doi:10.3389/fncel.2018.00533)
Supplement: TABLE S2 — List of cognate pheromone and kairomone receptors deorphanized in the Xenopus laevis and other functional bioassays. [file Table_2.pdf]

Supplementary table 2. List of cognate pheromone and kairomone receptors deorphanized in the *Xenopus laevis* and other functional bioassays.

Xenopus laevis expression system

Odorant receptor type	Receptor	Species	Ligand	EC50 (µM)	Reference
Pheromone receptor pheromone pairs	OR13-ORco	*Helicoverpa armigera*	Z11-16:Ald	3.4030	Liu et al., 2013
	OR01-ORco	*Plutella xyllostella*	Z11-16:Ald	0.8800	Mitsuno et al., 2008
	OR06-Orco	*Ostrinia nubilalis*	Z11-14:OAc	0.8600	Wanner et al., 2010
	OR10-ORco	*Amyelois transitella*	Z11Z13-16:Ald	0.499	Xu et al., 2012
	OR13-ORco	*Heliothis virescens*	Z11-16:Ald	0.3670	Wang et al., 2011
	OR11-ORco	*Apis mellifera*	9-oxo-2-decenoic acid	0.2800	Wanner et al., 2007
	OR04-ORco	*Plutella xyllostella*	Z9-14: OAc	0.2430	Sun et al., 2013
	OR13-ORco	*Heliothis virescens*	Z9-14:Ald	0.2400	Wang et al., 2011
	OR01-ORco	*Bombyx mori*	E10,Z12-16:OH (Bombykol)	0.0454	Xu et al., 2012
	OR03-ORco	*Ostrinia furnacalis*	Z11-14:OAc	0.0250	Leary et al., 2012
Kairomone receptors cognate ligand pairs	OR56-ORco	*Bombyx mori*	Z-jasmone	6.0000	Tanaka et al., 2009
	OR12-ORco	*Spodoptera litura*	Z3-6:OAc	0.3390	Zhang et al., 2013
	OR02-ORco	*Aedes aegypti*	Indole	0.3700	Bohbot et al., 2011
	OR03-ORco	*Spodoptera exigua*	E-b-farnesene	0.3000	Liu et al., 2014
	OR02-ORco	*Culex quinquefasciatus*	Indole	0.2800	Pelletier et al., 2010
	OR65-ORco	*Anopheles gambiae*	Eugenol	0.2220	Wang et al., 2010
	OR02-ORco	*Anopheles gambiae*	Indole	0.1670	Wang et al., 2010
	OR08-ORco	*Aedes aegypti*	(*R*)-(–)-1-octen-3-ol	0.1580	Bohbot et al., 2009
	OR10-ORco	*Aedes aegypti*	Skatole	0.1090	Bohbot et al., 2012
	OR10-ORco	*Culex quinquefasciatus*	Skatole	0.0900	Hughes et al., 2010
	OR9-ORco	*Aedes aegypti*	Skatole	0.0048	This study

Other expression system

Odorant receptor type	Receptor	Species	Ligand	Methodology	Reference
Kairomone receptors cognate ligand pairs	OR2-ORco	*Aedes albopictus*	Indole	Calcium imaging-HEK	Scialo et al., 2012
	OR10-ORco	*Anopheles sinensis*	Skatole	Calcium imaging-HEK	Liu et al., 2018
	OR2-ORco	*Anopheles gambiae*	Skatole	*Drosophila* empty neuron system	Liu et al., 2018
