# Supplementary material for: Supersensitive Odorant Receptor Underscores Pleiotropic Roles of Indoles in Mosquito Ecology
Source: Front Cell Neurosci. 2019 Jan 24;12:533. doi: 10.3389/fncel.2018.00533 (PMC6353850; doi:10.3389/fncel.2018.00533)
Supplement: TABLE S3 — Accession numbers of indolergic receptors used in Figure 3 (Ae., Aedes; Cu., Culex; An., Anopheles; To., Toxorhynchites; D., Drosophila). [file Table_3.pdf]

Supplementary table 3. Accession numbers of indolergic receptors used in figure 3 (*Ae.*, *Aedes*; *Cu.*, *Culex*; *An.*, *Anopheles*; *To.*, *Toxorhynchites*; *D.*, *Drosophila*).

Indolergic OR	Species	Accession number	Reference
OR2	*Ae. aegypti*	ACH69138	Bohbot et al., 2011
	Ae. albopictus	AEX65778	Scialo et al., 2012
	Cu. quinquefasciatus	ADF42901	Pelletier et al., 2010
	An. sinsensis	KFB39839	Zhou et al., 2014
	An. quadriannulatus	ACH69144	Bohbot et al., 2011
	An. gambiae	XP_310173	Holt et al., 2003
	An. funestus	AIO10776	Xu and Leal, 2014 (Unpublished)
	An. stephensi	ACH69149	Bohbot et al., 2011
	An. darlingi	ETN60992	Marinotti et al., 2013
	To. amboinensis	m.10291	Zhou et al., 2014
OR9	*Ae. aegypti*	ACH69140	Bohbot et al., 2011
	Ae. albopictus	KXJ75419	Chen et al., 2015
	Cu. quinquefasciatus	XP_001864543	Aktinson et al., 2007 (Unpublished)
OR10	*Ae. aegypti*	ACH69137	Bohbot et al., 2011
	Ae. albopictus	KXJ77288	Chen et al., 2015
	Cu. quinquefasciatus	ADF42902	Pelletier et al., 2010
	An. quadriannulatus	ACH69146	Bohbot et al., 2011
	An. gambiae	XP_310172	Holt et al., 2002
	An. stephensi	ACH69150	Bohbot et al., 2011
	An. sinsensis	KFB39838	Zhou et al., 2014
	An. darlingi	ETN65485	Marinotti et al., 2013
	To. amboinensis	m.26775	Zhou et al., 2014
OR43	*D. melanogaster*	AAF59173	Adams et al., 2000
